# Tomato and Olive Bioactive Compounds: A Natural Shield against the Cellular Effects Induced by β-Hexachlorocyclohexane-Activated Signaling Pathways

**DOI:** 10.3390/molecules26237135

**Published:** 2021-11-25

**Authors:** Elisabetta Rubini, Marco Minacori, Giuliano Paglia, Alberto Macone, Silvia Chichiarelli, Fabio Altieri, Margherita Eufemi

**Affiliations:** 1 Department of Biochemical Science “A. Rossi Fanelli”, Faculty of Pharmacy and Medicine, Sapienza University of Rome, P.le Aldo Moro 5, 00185 Rome, Italy; elisabetta.rubini@uniroma1.it (E.R.); marco.minacori@uniroma1.it (M.M.); giuliano.paglia@uniroma1.it (G.P.); alberto.macone@uniroma1.it (A.M.); silvia.chichiarelli@uniroma1.it (S.C.); margherita.eufemi@uniroma1.it (M.E.); 2 Enrico ed Enrica Sovena Foundation, 00199 Rome, Italy; 3 Fondazione Federico Calabresi Onlus, 00186 Rome, Italy

**Keywords:** environmental disease, cancer, environmental pollution, organochlorine pesticides, β-hexachlorocyclohexane, natural dietary compounds, signaling pathways

## Abstract

The β-isomer of hexachlorocyclohexane (β-HCH) is a globally widespread pollutant that embodies all the physicochemical characteristics of organochlorine pesticides, constituting an environmental risk factor for a wide range of noncommunicable diseases. Previous in vitro studies from our group disclosed the carcinogenic potential of β-HCH, which contributes to neoplastic transformation by means of multifaceted intracellular mechanisms. Considering the positive evidence regarding the protective role of natural bioactive compounds against pollution-induced toxicity, micronutrients from olive and tomato endowed with the capability of modulating β-HCH cellular targets were tested. For this purpose, the solution obtained from a patented food supplement (No. EP2851080A1), referred to as Tomato and Olive Bioactive Compounds (TOBC), was administered to the androgen-sensitive prostate cancer cells LNCaP and different biochemical and cellular assays were performed to evaluate its efficiency. TOBC shows a dose-dependent significant chemoprotection by contrasting β-HCH-induced intracellular responses such as STAT3 and AhR activation, disruption of AR signaling, antiapoptotic and proliferative activity, and increase in ROS production and DNA damage. These experimental outcomes identified TOBC as a suitable functional food to be included in a diet regimen aimed at defending cells from β-HCH negative effects, recommending the development of tailored enriched formulations for exposed individuals.

## 1. Introduction

The Global Environmental Changes (GEC) encompass climate change, loss of biodiversity and over-exploitation of ecosystems, resulting into a manifold negative impact not only on the entire planet but also on human health [1]. The so-called “civilization diseases”, in fact, are strictly related to the environmental dynamics and are often synonymous with noncommunicable diseases (NCDs), an umbrella term used to describe a heterogeneous group of pathologic conditions that are not transmissible through person-to-person contact. NCDs include a wide spectrum of health problems, such as cardiovascular and respiratory diseases, cancers, diabetes, and mental health conditions, together with neurologic, endocrine, gastrointestinal, renal, allergic, and autoimmune disorders [2].

The World Health Organization (WHO) estimates that NCDs are collectively responsible for almost 70% of all deaths worldwide and that as much as 24% of the global disease can be attributed to environmental risk factors [3]. Environmental diseases (ENVDs) are noncommunicable diseases affecting population long-term exposed to toxic chemicals [4,5,6,7]. The intensified incidence of ENVDs across the past two generations reached near pandemic levels and constitutes a public health emergency that demands an immediate intervention for decontaminating both the environment and biological systems.

In general, at environmental level, a remediation intervention consists in a series of measures aimed at removing or reducing the contamination sources, bringing the concentration of dangerous substances under the threshold levels. An environmental remediation project is mainly articulated in four steps:

1. Preliminary investigation, for the evaluation of site contamination;
2. Characterization, for the physicochemical characterization of pollutants;
3. Risk assessment, for the determination of the Risk Threshold Concentrations (RTC);
4. Remediation plan, for the implementation of site reclamation activities [8].

A parallel procedure may be extended to exposed population. The preliminary investigation should be epidemiological surveillance programs carried out to identify and characterize the hazardous compounds to determine their plasma concentration; then, the risk assessment should take into account the toxicity and the biomolecular mechanisms elicited by the contaminating agents, providing important elements to predict a possible link between the exposure and a range of health conditions. This operational scheme will be the backbone for the development of a “human body remediation”, allowing the finalization of a targeted therapeutic approach [9]. Among a variety of environmental toxic substances, β-hexachlorocyclohexane (β-HCH) is the organochlorine compound that better exemplifies all the specific features of persistent organic pollutants: it is hard to degrade and exhibits marked lipophilia; high bioaccumulation, with an estimated half-life of 7 to 10 years in humans [10]. Since β-HCH is a waste byproduct from the synthesis of technical grade lindane (γ-HCH) and does not have intrinsic insecticidal activity, the interest in this apparently trivial pollutant arises from its nature as a “fossil isomer” [11,12]. Compared with that of the other members of the hexachlorocyclohexane family, in fact, β-HCH shows an impressive chemical stability, resulting in a substantial environmental impact. Despite the production of technical lindane was discontinued in the 1970s, β-HCH still constitutes a heavy burden, accounting for 7.2 million tons illegally stocked worldwide [13]. In addition, significant exposure data for β-HCH are collected in a population-based epidemiologic study carried out during the three-year period 2012–2015 in the industrial district of Valle del Sacco, one of the 42 Italian contaminated Sites of National Interest (SIN) [14,15]. The information provided by this surveillance report fulfilled the requirements for β-HCH characterization and stimulated an in-depth investigation of the molecular derangements induced by this pollutant. The concentration extrapolated from β-HCH plasma levels detected in monitored individuals was, indeed, a starting point to explore the molecular mechanisms of this compound by reproducing the real exposure conditions [16]. Previous findings from our research group contributed to elucidate the intracellular targets of β-HCH, disclosing its involvement in neoplastic transformation. The biochemical pleiotropism of β-HCH reflects the substantial effects of this small molecule on cellular homeostasis. Results from experiments, performed on a panel of different cell lines, demonstrated that the oncoprotein STAT3 (Signal Transducer and Activator of Transcription 3) is a converging point in the transduction pathways triggered by β-HCH and outlined the xenobiotic functions of this organochlorine, which is capable of impacting on the three stages of carcinogenesis [17,18]. In particular, β-HCH was proven to: foster cell proliferation and clonogenic potential, act as an endocrine disruptor via Aryl hydrocarbon Receptor and Androgen Receptor activation (AhR and AR), induce Reactive Oxygen Species (ROS) production and DNA damage, promote the metabolic rewiring toward aerobic glycolysis (Warburg Effect) and affect cell cycle progression [19].

The overall knowledge about β-HCH available from experimental and epidemiological studies kindled the interest in developing a remediation step intended to prevent and mitigate β-HCH-induced toxicity. The most immediate and cost-effective strategy may be a nutritional approach consisting in the increased dietary uptake of natural-derived phytochemicals endowed with the capacity of modulating the cellular responses to β-HCH [20,21]; in the optical of what we could define as green therapy, the ideal candidates should be bioactive compounds for which the intracellular mechanisms of action were identified [22,23]. The dietary elements that better fit with this purpose are those contained in tomato and olives, the undisputed protagonists of the Mediterranean diet: the winning combination between their easily availability and rich content of bioactive molecules, in fact, is consistent with the concept of green therapy [24]. Tomato and olives, either as they are or as food supplements, revealed to have important health benefits, which are supported by scientific literature; in particular, most of the active principles contained in tomato and olives (i.e., lycopene, tocopherol, tyrosol) are associated with reduced cardiovascular risk, positive impact on cancer, stimulation of the immune system, antibacterial and antiviral properties [25,26]. Considering that the molecular players responsible for β-HCH effects (STAT3, AR and AhR, ROS) are multitasking in terms of biological functions, they could constitute suitable targets to evaluate the translational protective role of tomato and olives-derived phytocompounds toward β-HCH intracellular activities [27,28]. The presented cellular study is aimed at providing a defense tool against β-HCH harmfulness, as well as an experimental design which can be extended to other widely diffused pollutants to propose targetable biomolecules and a nutraceutical support within a preventive and therapeutic framework for oncologic patients exposed to those substances. 

## 2. Materials and Methods

### 2.1. Extraction of Tomato and Olive Bioactive Compounds (TOBC)

Bioactive compounds were obtained from a food supplement registered by the Italian Health Ministry (patent No. EP2851080A1) based on spray-dried whole tomatoes and olive mill wastewater. The patented food supplement has been kindly provided by Janus Pharma (Rome, Italy). The lipophilic solution obtained from the supplement is referred to as TOBC (Tomato and Olive Bioactive Compounds). Essentially, 1 g of the food supplement powder was dissolved in 10 mL of DMSO: the organic solvent was used to make available to cells as much active molecules as possible. The composition of the patent No. EP2851080A1 was confirmed by GC-MS-analysis: the bioactive compounds present in the TOBC solution and their respective concentrations are listed in Table 1. Chromatograms are reported in Supplementary Material (Figure S1).

### 2.2. GC-MS Analysis

Untargeted metabolomic analysis of TOBC solution was carried out by GC-MS according to Pintus et al. Ref. [29]. The extract was analyzed as such or after TBDMS derivatization. Briefly, a 20 μL aliquot of the extract was dried, and 50 μL of neat MTBSTFA and 50 μL of acetonitrile, were added. The mixture was heated at 80 °C for 1 h. The sample was dried under a stream of N2 and the residue was resuspended in 0.1 mL of dichloromethane.

GC-MS analyses were performed with an Agilent 7890B gas chromatograph coupled to a 5977B quadrupole mass selective detector (Agilent Technologies, Palo Alto, CA, USA). Chromatographic separations were carried out with an Agilent HP5ms fused-silica capillary column (30 m × 0.25 mm i.d.) coated with 5%- phenyl-95%-dimethylpolysiloxane (film thickness 0.25 μm) as stationary phase. Injection mode: splitless at a temperature of 280 °C. Column temperature program: 70 °C (1 min) then to 300 °C at a rate of 20 °C/min and held for 10 min. The carrier gas was helium at a constant flow of 1.0 mL/min. The spectra were obtained in the electron impact mode at 70 eV ionization energy; ion source 280 °C; ion source vacuum 10–5 Torr. MS analysis was performed simultaneously in TIC (mass range scan from *m*/*z* 50 to 600 at a rate of 0.42 scans s-1). The untargeted metabolomic analysis of TOBC solution was performed by comparison with the fragmentation profiles of the NIST2017 database.

### 2.3. Cell Culture

Human prostate epithelial cell line LNCaP was obtained from American Type Culture Collection (ATCC). Cells were grown to 80% confluence at 37 °C in 5% CO_2_ in the appropriate culture medium, RPMI 1640 (Sigma-Aldrich, Milano, Italy, cat. R0883,) supplemented with 1% sodium pyruvate (Sigma-Aldrich, Milano, Italy, cat. S8636), 10% fetal bovine serum (Sigma–Aldrich, Milano, Italy, cat. F7524), 2 mM glutamine, 100 μg/mL streptomycin and 100 U/mL penicillin (Sigma-Aldrich, Milano, Italy, cat. P4333). TOBC was tested on LNCaP cells at a final concentration of 1 mg/mL and β-hexachlorocyclohexane (β-HCH) (Sigma-Aldrich, Milano, Italy, 33376) was used a final concentration of 10 μM. For the inhibition of AR and AhR were used, respectively, 120 nM bicalutamide (Sigma-Aldrich, Milano, Italy, cat. B9061) and 150 nM CH223191 (Sigma-Aldrich, Milano, Italy, cat. C8124).

### 2.4. Cell Viability

The impact of β-HCH and TOBC on cell viability was evaluated by seeding 12,000 cells/well in 96-well plates. Cells were pretreated 3 h with TOBC 1 mg/mL and then stimulated with 10 μM β-HCH for 48 h. Cell viability was measured using MTT (3-(4,5-dimethylthiazol-2-yl)-2,5-diphenyl-2H-tetrazolium bromide) (Sigma-Aldrich, Milano, Italy, cat. M2128). Briefly, the culture medium was removed and 125 μL of MTT solution (0.5 mg/mL MTT in culture medium) was added to each well. After 3 h incubation, the solution was removed and the insoluble formazan dye, resulting from the conversion of tetrazolium salt by metabolically active cells, was dissolved by adding 125 μL/well of DMSO and measured at 570 nm using the Appliskan plate reader (Thermo Fisher Scientific, Monza, Italy).

### 2.5. Protein Extraction and Immunoblotting

Protein extraction and immunoblotting analysis were performed essentially according to Rubini et al. [19]. Cells cultured on 6-well plates at a density of 300,000 cells/well, harvested by centrifugation, and washed in PBS (Sigma–Aldrich, Milano, Italy, cat. D8662). Total protein extracts were obtained using a lysis buffer containing 2% SDS (BioRad, Segrate, Italy, cat. 161030), 20 mM Tris-hydrochloride pH 7.4 (Sigma–Aldrich, cat. T3253), 2 M urea (Sigma–Aldrich, Milano, Italy, cat. U5378), 10% glycerol (Merck, Milano, Italy, cat. GE17-1325-01) added with 2 mM sodium orthovanadate (Sigma–Aldrich, Milano, Italy, cat. S6508) 10 mM DTT (Sigma–Aldrich, Milano, Italy, cat. D9779), and a protease inhibitors cocktail diluted 1:100 (Immunological Sciences, Roma, Italy, cat. IK-96010). Proteins were resolved by SDS-PAGE 10% TGX FastCastTM Acrylamide gel (BioRad, Segrate, Italy, cat. 161-0183) and transferred on PVDF membranes using Trans-Blot^®^ TurboTM Transfer System (BioRad, Segrate, Italy, cat. 170-4247). The membranes were blocked with 3% BSA (Immunological Sciences, Roma, Italy, cat. ISP6154-100) or 0.2% *w*/*v* I-block (Thermo Fisher Scientific, Monza, Italy, cat. T2015) in Tris-buffered saline containing 0.05% Tween-20 (Sigma–Aldrich, Milano, Italy, cat. P7949) (TBS-T) and incubated with a specific primary antibody for 1 h. Subsequently, membranes were washed three times in TBS-T, and then incubated for an additional hour with phosphatase-conjugated secondary antibody (Sigma–Aldrich, cat. A3687-A3688, dilution 1:5000). The alkaline phosphatase signal was detected with BCIP/NBT reagents (Carl Roth, Milano, Italy, cat. 6368.1 and 4421.3). The intensity of protein bands was quantified using the ImageLab Software. Membrane images were acquired by Molecular Imager^®^ ChemiDoc™ MP System (Bio-Rad, Segrate, Italy), and the intensity of protein bands was quantified using the ImageLab Software. The immunoblotting detection was carried out using specific primary antibodies: anti-pY705STA3 (Cell Signaling, Pero, Italy, cat. D3A7), anti-STAT3 (Cell Signaling, Pero, Italy, cat. 124H6), anti-pS139H2AX (Santa Cruz Biotechnology, Segrate, Italy, cat. 101696), anti β-actin (Sigma–Aldrich, Milano, Italy, cat. A1978 clone AC-15) diluted according to manufacturer’s instruction depending on the experiment. 

### 2.6. Immunofluorescence

Immunofluorescence analysis was performed essentially according to Cocchiola et al. [30]. Cultured cells were grown on coverslips and pre-treated 4 h with TOBC 1 mg/mL and then stimulated for 4 h with 10 μM β-HCH. Cells grown on coverslips were washed with PBS, fixed with 4% formaldehyde for 15 min, and then rinsed with PBS (Sigma–Aldrich, Milano, Italy, cat. D8662). Cells were permeabilized with cold methanol (−20 °C) for 5 min. After washing three times with PBS, the cells were blocked overnight with 3% *w*/*v* BSA (Immunological Sciences, Roma, Italy, cat. ISP6154-100) in PBS. Fixed cells were processed by immunofluorescence staining using specific primary antibodies against Androgen receptor (Cell Signaling, Pero, Italy, cat. D6F11) against Aryl hydrocarbon receptor (Invitrogen, Monza, Italy cat. MA1-514) and against STAT3 (Cell Signaling, Pero, Italy, cat. 124H6) properly diluted in PBS containing 2% *w*/*v* BSA for 1 h. Following three washes with PBS added to with 0.05% Triton and 2% *w*/*v* BSA (PBS-T), cells were incubated for 1 h in the darkness with a FITC-conjugated secondary antibody (Jackson Immunoresearch, AlexaFluor 488-conjugated, Cambridge, UK, cat. 211-545-109, dilution 1:800). Cell nuclei were counterstained with 100 ng/mL Hoechst (Sigma–Aldrich, Milano, Italy, cat. 94403) for 15 min. After washing with PBS-T, coverslips were mounted on glass microscope slides with DuolinkTM Mounting Medium and examined using a fluorescence microscope (Leica AF6000 Modular System, Leica, Milano, Italy) with 63× oil immersion objective. Samples were captured under the same acquisition parameters and were background subtracted before analysis. Fluorescence intensity was quantified averaging across the CTCF (Corrected Total Cell Fluorescence) calculated via ImageJ on the same number of cells for both control and treated sample from different images according to McCloy et al. [31]. 

### 2.7. Reactive Oxygen Species (ROS) Detection

To evaluate antioxidant activity of TOBC versus Reactive oxygen species (ROS) generated by β-HCH, LNCaP were pretreated 3 h with TOBC 1 mg/mL and then were stressed with 10 μM β-HCH for 6 h or 75 μM tBHP for 1 h as a positive control. ROS were quantified using the CellROX Green Flow Cytometry Assay Kit (Thermo Fisher Scientific, Monza, Italy, cat. C10492) following the manufacturer’s instructions. Samples were analyzed by a fluorescent microscope Leica AF6000 Modular System (Leica, Milano, Italy) with 20× objective. 

### 2.8. Determination of Apoptosis

Cells were pretreated 3 h with TOBC solution 1 mg/mL and then were stimulated for 24 h with 10 μM β-HCH and 10 μM camptothecin (Sigma–Aldrich, Milano, Italy, cat. 208925) as a positive control. Apoptosis was determined using Annexin V-FITC (Immunological Sciences, Roma, Italy, cat. IK-11120) according to the manufacturer’s instructions. Samples were analyzed using a BD Accuri C6 flow cytometer (BD Biosciences, Milano, Italy) and a fluorescent microscope (Leica AF6000 Modular System) with 63× oil immersion objective. 

### 2.9. Cell Cycle Analysis

To analyze cell cycle by flow cytometry, cells were pretreated 3 h with TOBC 1 mg/mL and then stimulated with 10 μM β-HCH for 24 h. Then, after detaching them with trypsin, cells were washed with HBSS (Sigma–Aldrich, Milano, Italy, cat. 55021C) and fixed with 70% cold ethanol (Fluka, cat. 02860). Ethanol was added drop wise to the pellet while mixing by inversion. Samples were fixed to 30 min at 4 °C and then washed twice in HBSS. RNAse (Sigma–Aldrich, Milano, Italy, cat. R6513) was then added at a final concentration of 0.2 mg/mL and incubated for 5 min at 37 °C. Then, 60 μg/mL final concentrated propidium iodide (Sigma–Aldrich, Milano, Italy, cat. P4170) was added and samples were incubated for 45 min at 37 °C in the dark. Before flow cytometry analysis, samples were centrifuged and resuspended in the proper volume of HBSS. Samples were analyzed using a BD Accuri C6 flow cytometer (BD Biosciences, Milano, Italy). Results were analyzed using the ModFit LT software. 

### 2.10. Comet Assay

LNCaP cells were seeded on a 6-well plate at a density of 40,000 cells/well and were pretreated 3 h with TOBC 1 mg/mL and then incubated with 10 μM β-HCH for 6 h; cells stimulated with 75 μM tert-butyl hydroperoxide for 1 h were used as a positive control for DNA damage. Then, alkaline comet assay was performed essentially according to Yang et al. [32]. Briefly, slides were preliminarily coated with 1% agarose (Sigma–Aldrich, Milano, Italy, cat. A0576) and were let air-dry overnight. LNCaP cells were digested in trypsin for 3 min and trypsin was neutralized using the proper volume of RPMI medium. After centrifugation, cells were resuspended at a ratio 1:10 (*v*/*v*) in 1% low melting point agarose (Millipore, cat. 2070 OP) and 20 μL of the cells/agarose mixture were pipetted onto a slide and incubated overnight in the lysis solution (LS) (2.5 NaCl, 100 mM EDTA, 10 mM Tris-base, 200 mM NaOH, 1% sodium lauryl sarcosinate and 1% Triton X-100) at 4 °C. Then, slides were immersed for 1 h at 4 °C in the dark in Alkaline Electrophoresis Solution pH > 13 (AES) (200 mM NaOH, 1 mM EDTA) to allow DNA unwinding. Electrophoresis was carried out at 30 mA for 1 h at 4 °C. Then, slides were rinsed twice with dH_2_O and were immersed in 70% EtOH for 30 min at room temperature. After drying the slides for 15 min at 37 °C in the dark, slides were stained with propidium iodide (Sigma–Aldrich, Milano, Italy, cat. P4170) at a final concentration of 5 μM/mL (15 min at room temperature); then slides were rinsed with dH_2_O and dried for the acquisition. Images were captured using a fluorescence microscope (Leica AF6000 Modular System) with 20× objective and analyzed using the CaspLab software.

### 2.11. Colony Formation Assay

LNCaP cells were seeded at a density of 200 cells/mL in a 6-wells plate and pretreated for one week with 10 μM β-HCH and for further 5 days with TOBC 1 mg/mL. Medium was removed, cells were rinsed with PBS and fixed with cold MeOH for 30 min at 4 °C. Then, colonies were stained by incubating cells with a mixture of 1% crystal violet in 25% MeOH for 1 h at room temperature. After removing the staining solution, the plate with colonies was rinsed with abundant dH_2_O and was air-dried at room temperature. Colonies were counted using the ImageJ software according to Cai et al. [33].

### 2.12. Statistical Analysis

The repeatability of results was confirmed by performing all experiments at least three times. The obtained values are presented as mean and standard deviation. Statistical analysis was performed with GraphPad Prisma software using a Student’s *t*-test, one-way ANOVA test with Dunnet’s posthoc test and two-way ANOVA test with Bonferroni’s posthoc test.

## 3. Results

In the context of a cellular study intended for the development of a green dietary strategy against environmental pollution damage, the cell line choice represents a fundamental step of the experimental design. Selection criteria shall take into account the intracellular mechanisms regulating the molecular responses to β-HCH, which include STAT3 activation, endocrine system disruption, and oxidative stress induction.

On the basis of these biochemical requirements, the best candidate is represented by the hormone-responsive prostate cancer cell line LNCaP (AR+), which was already employed in our previous in vitro studies [17,19]. On the other hand, suitable bioactive compounds must exhibit a good modulatory activity toward β-HCH targets and have to be easily introduced in a balanced and healthy dietary regimen, such as the Mediterranean diet. The Mediterranean diet is not only a unique dietary pattern, but also a precious medical tool: greater adherence to the Mediterranean diet regimen, in fact, was associated with significant benefits with respect to certain cardiovascular risks, neurological disorders, metabolic syndromes and cancer [34,35]. The positive effects on human health strongly correlate with a wide array of beneficial nutrients and antioxidants commonly present in some of the staple components of the Mediterranean diet, such as olives and tomato. Therefore, our investigation took advantages from a patented food supplement enriched in highly bioavailable active compounds (patent No. EP2851080A1) derived from the combination of spray-dried whole tomatoes and a small percentage olive mill wastewater (98:2 ratio). The supplement was shown to possess potent antioxidant and anti-inflammatory activity in animal model [36] and human disease [37]. On the basis of its varied nutraceutical composition, this supplement could be a valid functional food to be included in a green diet plan; in addition, the employment of tomato scraps and olive oil production wastes gives a nod to the environment, allowing the recovery of potential contamination sources. In this study, the patent food supplement, hereafter referred to as TOBC (Tomato and Olives Bioactive Compounds), was solubilized and chemically characterized. TOBC solution mainly contains molecules that were shown to modulate β-HCH intracellular targets (Table 1).

### 3.1. TOBC Exhibits a Protective Role against Cancer Cells Proliferative Activity

**Figure 1:**
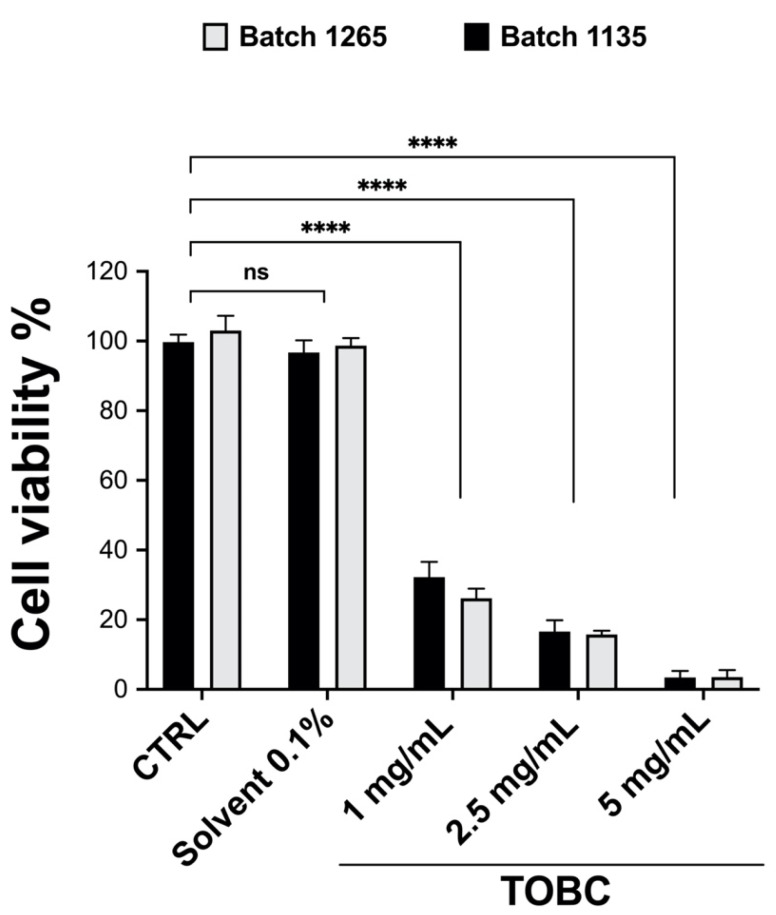
MTT assay performed on untreated LNCaP cells incubated for 48 h with TOBC solution (concentration ranging from 1 mg/mL up to 5 mg/mL) obtained from two different batches (No 1135 and No 1265). Impact of TOBC solutions on cell viability is comparable between two batches. Experiments were repeated three times with similar results. Statistical analyses were performed by two-way ANOVA using Bonferroni’s posthoc test, and obtained data are presented as mean ± SEM. The ANOVA test reveals that there is not a statistically significant interaction between the batches and the cell treatment (F(4,10) = 1.516; *p* = 0.2700), whereas TOBC concentration showed a statistically significant effect on cell viability (F (4,10) = 1009; *p* < 0.0001). Statistically significant differences (**** *p* < 0.0001) are marked with asterisks and are referred to the control.

To evaluate the potential anticancer effects of TOBC and to verify whether the solutions obtained from two batches of the supplement powder (No. 1,135 and No. 1,265) show reproducible efficiency, an MTT assay was performed on untreated LNCaP cells. As reported in Figure 1, TOBC is able to reduce the viability of the androgen sensitive (AR^+^) prostate cancer cells LNCaP by almost 70% after 48 h, suggesting TOBC antitumoral activity. Considering that the two batches show comparable effects in reducing cell proliferation, the batch No. 1265 at the lowest concentration (1 mg/mL) was selected for the subsequent experiments. 

**Figure 2:**
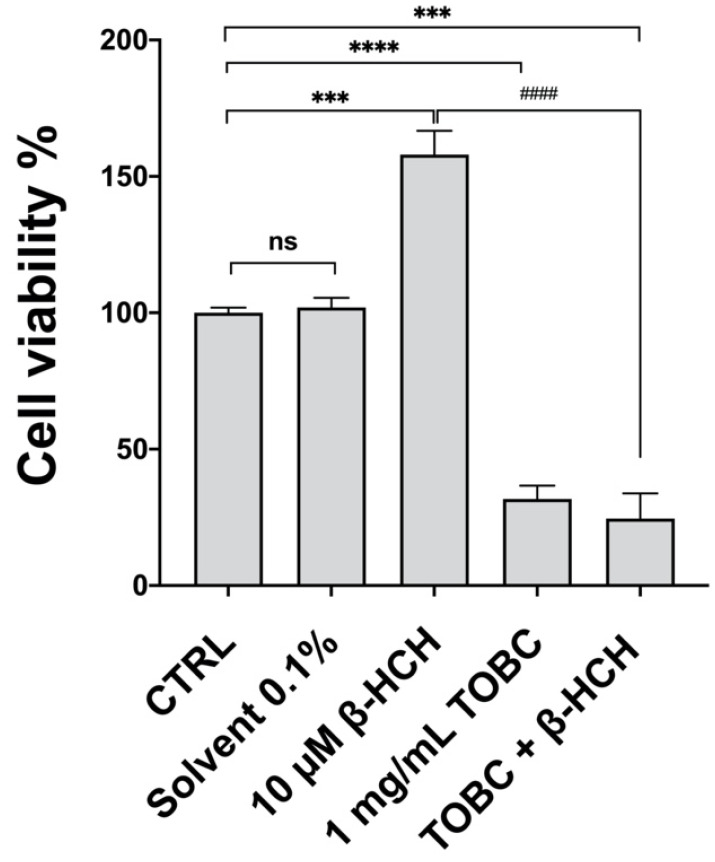
MTT assay on LNCaP cells pretreated for 3 h with 1 mg/mL TOBC solution, and then incubated for 48 h with 10 μM β-HCH. Experiments were repeated three times with similar results and obtained values are reported as mean and standard deviation. Statistical analysis was performed with GraphPad Prisma software using Student’s *t*-test. Statistically significant differences referred to control are marked with asterisks (*** *p* < 0.001; **** *p* < 0.0001); statistically significant differences between β-HCH and TOBC + β-HCH samples are marked with hashtags (^####^ *p* < 0.0001).

After that, the defensive role of TOBC against β-HCH was assessed. Untreated LNCaP cells were preincubated for 3 h with 1 mg/mL TOBC and then with 10 µM β-HCH for 48 h. β-HCH working concentration is the same already used in our previous cellular studies and was extrapolated by averaging across the plasma concentration values detected in exposed population under epidemiological surveillance. Results reported in Figure 2 evidence that TOBC counteracts the significant increase in cell viability induced by β-HCH, exerting a protective cytotoxicity either administered alone or in cotreatment with β-HCH: in the presence of TOBC, in fact, the percentage of vital cells is approximately 30%. These outcomes provide a first hint on the possible benefits of bioactive molecules present in TOBC. 

### 3.2. TOBC Inhibits STAT3 Pathway

**Figure 3:**
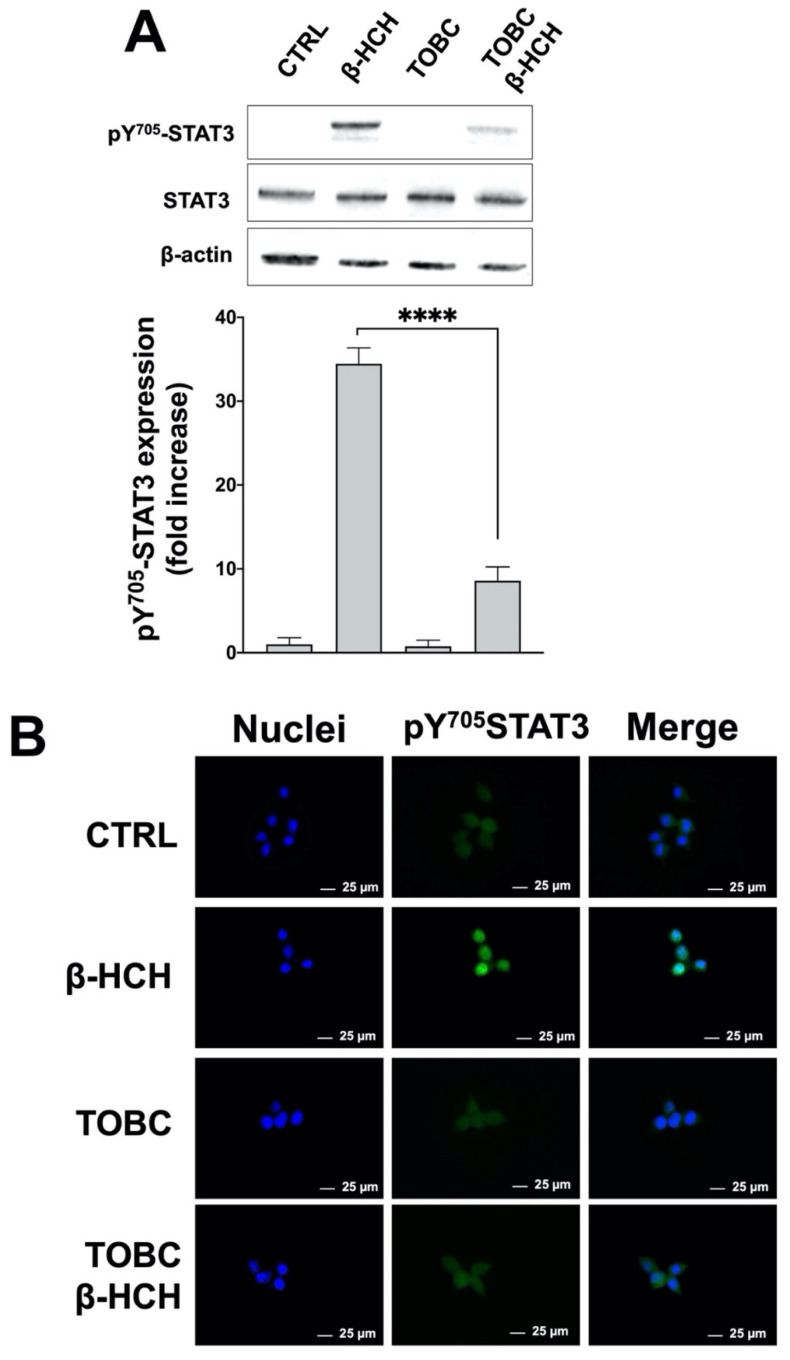
Immunoblotting and immunofluorescence were performed to evaluate activation of STAT3 in LNCaP cells pretreated for 3 h with 1 mg/mL TOBC, and then for 4 h with 10 μM β-HCH. (**A**) Total protein extracts were subjected to Western Blot analysis. Immunoblot evidenced STAT3 phosphorylation (pY705-STAT3) upon treatment with β-HCH, whereas a decrease in band intensity occurs in sample pretreated with TOBC. Phosphorylation levels were referred to amount of total STAT3 present in each sample and were compared to control. Experiments were repeated three times with similar results and obtained values are reported as mean and standard deviation. Statistical analysis was performed with GraphPad Prisma software using Student’s *t*-test. Statistically significant differences (**** *p* < 0.0001) are marked with asterisks and are referred to the control. (**B**) Cellular distribution of STAT3 followed by immunofluorescence in LNCaP cells. Images clearly show STAT3 nuclear localization in cells treated with β-HCH, but not in presence of TOBC. Selected images reported are representative of three independent experiments and were captured under same acquisition parameters. CTRL: Control untreated cells; β-HCH: Cells subjected to a 4 h stimulation with 10 µM β-HCH; TOBC: cells pretreated for 3 h with 1 mg/mL TOBC; TOBC+ β-HCH: cells pretreated for 3 h with 1 mg/mL TOBC and then for 4 h with 10 µM β-HCH.

The nongenotoxic carcinogenic mechanisms of organochlorine pollutants are attributable to their ability to affect transduction pathways crucially involved in the maintenance of cellular homeostasis. As described in our previous studies, one of the most relevant effects of β-HCH is the activation of STAT3, which modulates the intracellular response to many different stimuli (i.e., cytokines, growth factors, oxidative stress). STAT3 exerts its canonical transcriptional functions through the phosphorylation at the tyrosine residue 705 (pY705-STAT3), which drives protein homodimerization and its subsequent translocation into the nuclear compartment. STAT3 can be phosphorylated by an array of membrane receptors or cytoplasmic tyrosine kinases; in LNCaP cells, β-HCH was found to activate STAT3 via the SRC/STAT3 axis [17]. As a transcription factor, STAT3 directly regulates the expression of a wide range of oncogenic genes [61]. Taking into account that a good part of TOBC components is active towards STAT3 [62], it is conceivable to hypothesize that TOBC could counteract β-HCH-induced STAT3 phosphorylation and nuclear translocation. To determine the impact of TOBC on STAT3 activation, LNCaP cells were pretreated with 1 mg/mL TOBC solution for 3 h and then exposed to 10 µM β-HCH for 4 h. After total protein extraction, samples were subjected to western blot. As reported in Figure 3A, STAT3 phosphorylation occurs upon β-HCH stimulation, as previously described, but is considerably reduced by TOBC cotreatment; no pY705-STAT3 is detectable in the control and TOBC alone. The histogram displays the significant variation in pY705-STAT3 expression levels determined by densitometric analysis. To further confirm STAT3 activation, its cellular localization was followed by immunofluorescence performed under the same aforementioned experimental conditions. Figure 3B clearly indicates that STAT3 is present in the nuclei of LNCaP treated with β-HCH, whereas it is not observable in the control sample and in the presence of TOBC, neither alone nor in cotreatment β-HCH. These results support the capability of TOBC to inhibit STAT3 activation.

### 3.3. TOBC Inhibits AhR Activation

**Figure 4:**
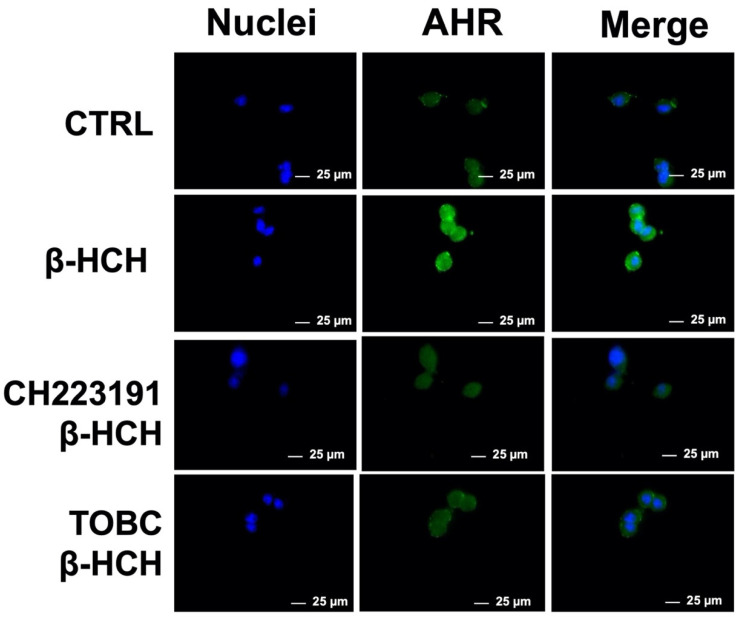
Cellular distribution of AhR followed by immunofluorescence in LNCaP cells. As displayed in the figures, receptor is only present in nuclei of cells treated with β-HCH alone, but not in presence of CH223191 nor TOBC. Selected images are representative of three independent experiments and were captured under same acquisition parameters CTR: control untreated cells; β-HCH: cells subjected to a 4 h stimulation with 10 µM β-HCH; β-HCH + CH223191: cells after 2 h pre-incubation with 150 nM CH223191, followed by 4 h of 10 µM β-HCH stimulation; TOBC+ β-HCH: cells pretreated 3 h with 1 mg/mL TOBC, and then for 4 h with 10 µM β-HCH. CH223191 was used as a positive control for AhR inhibition.

Exogenous substances that come into contact with the human body are referred to as xenobiotics and include a wide range of non-physiological and structural divergent chemicals, comprising organochlorine pollutants. Organochlorine pollutants may bind and modulate several endocrine receptors, one for all the Aryl Hydrocarbon Receptor (AhR). AhR is considered the xenobiotic sensor par excellence and constitutes a converging point for many physiological intracellular processes [63]. From a biochemical point of view, AhR is a cell nuclear receptor that acts as ligand-activated transcription factor involved in the recognition and metabolism of xenobiotics, leading to the expression of enzymes needed for their clearance from the body. Previous data demonstrated that AhR constitutes one of the molecular targets of β-HCH, which induces receptor nuclear localization [19]. To verify its inhibition by TOBC, AhR cellular localization was followed by immunofluorescence. LNCaP were incubated for 3 h with 1 mg/mL TOBC solution and subsequently treated with 10 µM for 4 h. Images show that TOBC is able to block AhR nuclear translocation, which instead occurs when cells are exposed to β-HCH alone (Figure 4). To corroborate this result, cells were also treated with β-HCH following a preincubation with the AhR antagonist CH223191 [64] at a final concentration of 150 nM, which was already proven to block β-HCH-dependent AhR activation.

### 3.4. TOBC Interfere with β-HCH Endocrine Disrupting Activity

**Figure 5:**
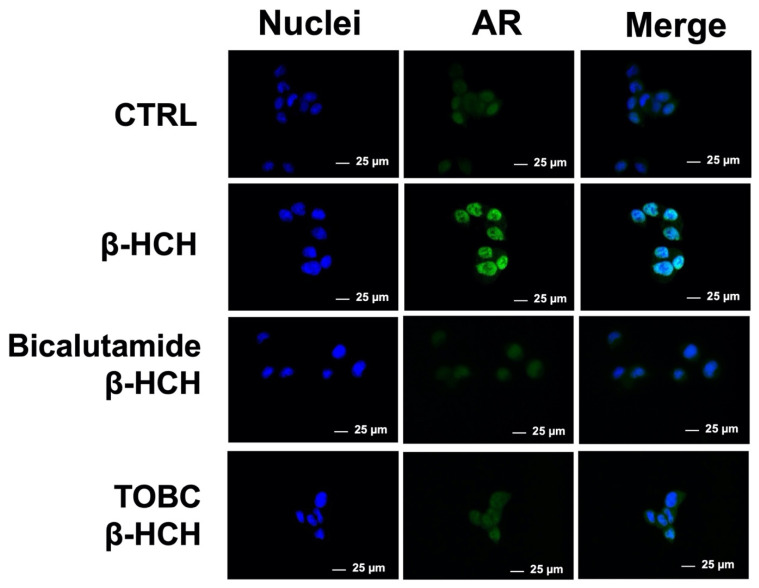
Cellular distribution of AR followed by immunofluorescence in LNCaP cells. β-HCH induces AR nuclear localization, which is inhibited by both TOBC and bicalutamide. Selected images are representative of three independent experiments and were captured under same acquisition parameters. CTR: control untreated cells; β-HCH: cells subjected to a 4 h stimulation with 10 µM β-HCH; β-HCH + Bicalutamide: cells pretreated overnight with 120 nM Bicalutamide and then subjected to a 4 h stimulation with β-HCH; TOBC+ β-HCH: cells pretreated 3 h with TOBC 1 mg/mL, and then for 4 h with 10 µM β-HCH. Bicalutamide was used as a positive control for AR inhibition.

Some organochlorines have physicochemical properties similar to those of natural circulating hormones and are able to mimic or block hormone-related activities, leading to an imbalance in cellular homeostasis; compounds exhibiting these features are classified as Endocrine Disrupting Chemicals (EDCs). β-HCH was already proven to act as an EDC by driving AR nuclear translocation and transcriptional activation in the AR-positive prostate cancer cell line LNCaP [19]. Considering the elicitation of AR signaling cascade in response to β-HCH, it would appear reasonable to investigate whether TOBC could interfere with this mechanism. As previously described for the determination of AhR activation, cells were subjected to immunofluorescence to evaluate AR nuclear localization after 4 h of exposition to β-HCH following a 3 h pretreatment with TOBC. Figure 5 provides unconfutable evidence that TOBC suppresses AR nuclear translocation, corroborating its protective potential toward β-HCH endocrine disrupting activity. To further support this result, cells were treated with β-HCH in the presence of the chemotherapeutic agent Bicalutamide (120 nM), used as a positive control for AR inhibition [65].

### 3.5. Protective Activity of TOBC towards Oxidative Stress

**Figure 6:**
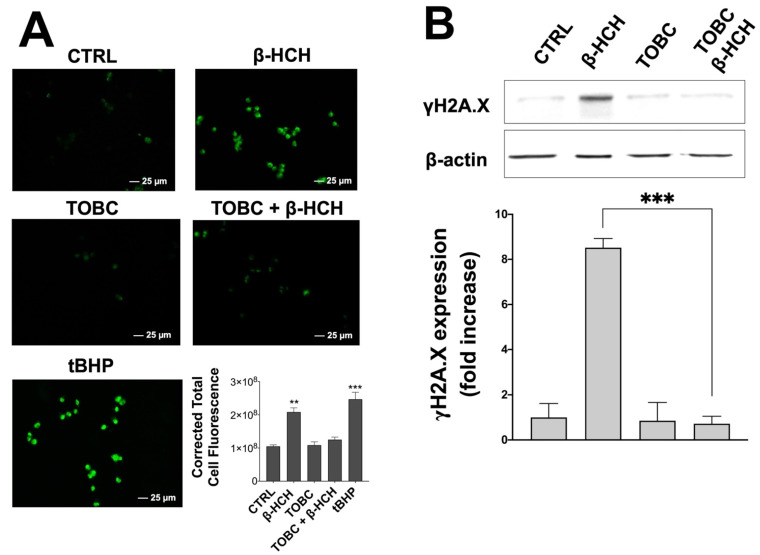

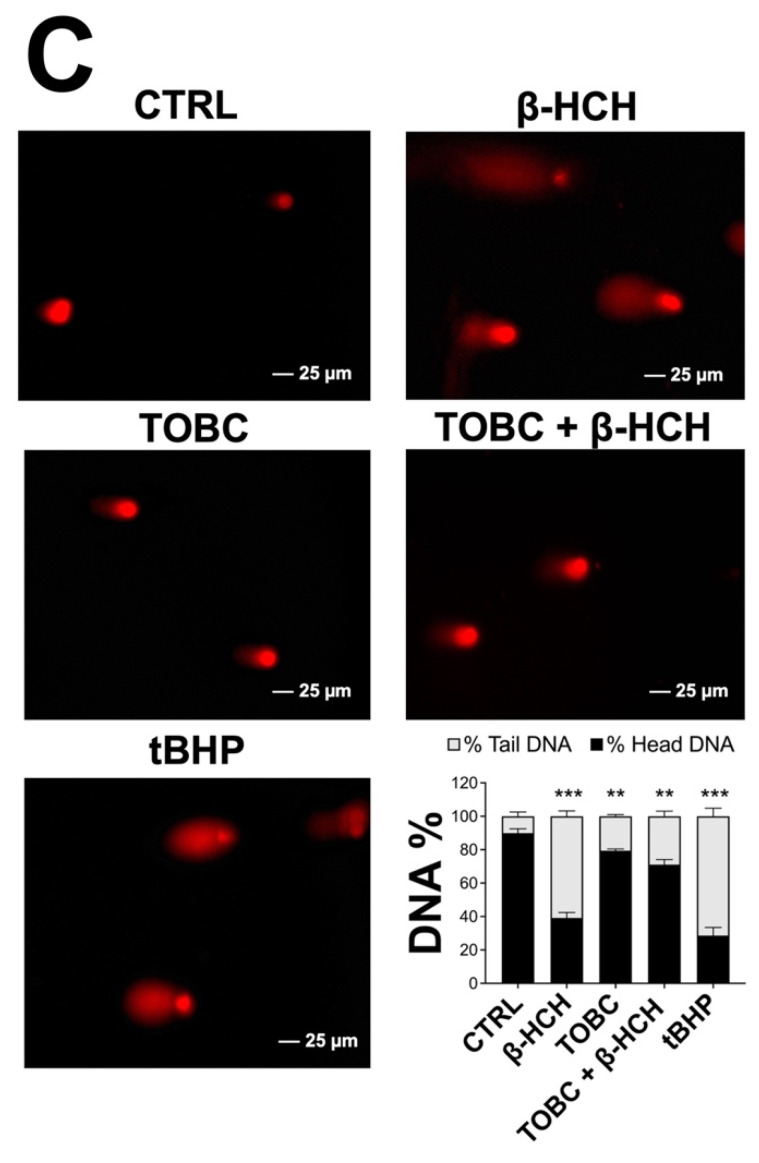
Immunofluorescence and immunoblotting were carried out to determine TOBC protective activity towards oxidative stress. (**A**) TOBC exhibits antioxidant activity versus reactive oxygen species (ROS) generated by β-HCH. Images and relative fluorescence quantification reported in histogram clearly show a reduction of ROS in presence of TOBC; treatment with 75 µM tBHP for 1 h was employed as a positive control for ROS production. Fluorescence intensity was quantified by averaging across CTCF (Corrected Total Cell Fluorescence) calculated with ImageJ on same number of cells from different images of each sample. Images were captured under same acquisition parameters and are representative of three independent experiments. (**B**) Immunoblot analysis of γH2A.X on total protein extracts obtained from LNCaP cells. Figure clearly shows that TOBC reduces H2A.X phosphorylation induced by β-HCH. Phosphorylation levels were measured on amount of β-actin present in each sample and were referred to control. (**C**) DNA damage was evaluated using Comet Assay. As shown in figure and confirmed by histogram, DNA fragmentation caused by β-HCH is reduced in presence of TOBC; 75 µM tBHP for 1 h was employed as a positive control for DNA fragmentation. Extent of DNA fragmentation was assessed by quantifying percentage of DNA in comet tail using Casp Lab software. Experiments were repeated three times with similar results and obtained values are presented as mean and standard deviation. CTRL: control (untreated cells); β-HCH: cells subjected to 6 h stimulation with 10 µM β-HCH; TOBC: Cells subjected to a 3 hours’ stimulation with TOBC 1 mg/mL; TOBC + β-HCH: cells pretreated 3 h with TOBC 1 mg/mL and then for 6 h with 10 µM β-HCH. Statistical analysis was performed with GraphPad Prisma software using Student’s *t*-test. Statistically significant differences (* *p* < 0.05; ** *p* < 0.005; *** *p* < 0.001) are marked with asterisks and are referred to control.

The genotoxicity of β-HCH is correlated to its capability of promoting oxidative stress via ROS production. ROS increase is probably dependent on a sustained cellular proliferative activity as a consequence of the release of Epidermal Growth Factor (EGF) in response to β-HCH, as described in a previous work [18]. Since the principal components of TOBC are well-established antioxidants, variations in ROS levels were determined by immunofluorescence using the CellROX reagent. Cells stimulated with tert-butyl hydroperoxide (tBHP) were taken as a positive control. As displayed in Figure 6A, an increase in fluorescence intensity occurs upon both β-HCH and tBHP treatment, whereas TOBC seems to act as a scavenger of β-HCH-induced ROS (experimental details are reported in figure caption). This result is further highlighted in the histogram (Figure 6A).

On the basis of this evidence, TOBC was tested on LNCaP cells to explore its protective role against β-HCH genotoxicity. Firstly, the phosphorylation status of the histone H2A.X (γH2A.X) was evaluated: the phosphorylation of histone H2A.X at serine 139, in fact, is a post-translational modification that constitutes a solid and versatile endpoint to investigate the genotoxic potential of a substance [66]. 

Total protein extracts from LNCaP cells incubated for 6 h with β-HCH, following or not a 3 h pretreatment step with TOBC, were subjected to immunoblotting using a specific antibody against γH2A.X; 75 µM tert-butyl hydroperoxide for 1 h (tBHP) was taken as a positive control. The resulting blot and the relative densitometric analysis (Figure 6B) show an increased H2A.X phosphorylation in response to β-HCH, whereas low and comparable γH2A.X expression levels are evident in the control and in cells treated with TOBC, either alone or together with β-HCH. 

To confirm the correspondence between H2A.X phosphorylation and DNA damage, comet assay was carried out on LNCaP cells exposed to β-HCH with or without a preincubation with TOBC, accordingly to the same aforementioned experimental setting; tBHP was used as a positive control for DNA fragmentation. As evident in Figure 6C, TOBC in association with β-HCH substantially reduces the extent of DNA fragmentation visualized by the comet tail induced by β-HCH treatment alone. 

Taken all these data into account, together with the molecular effects on signaling pathways, TOBC seems even more to be a promising supplement to be included in an adjuvant diet plan.

### 3.6. TOBC Effects on Cell Cycle Distribution and Apoptosis

**Figure 7:**
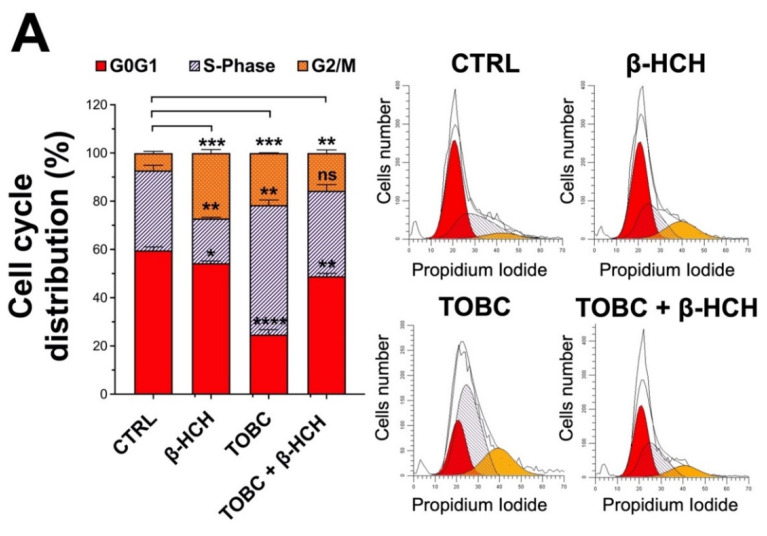

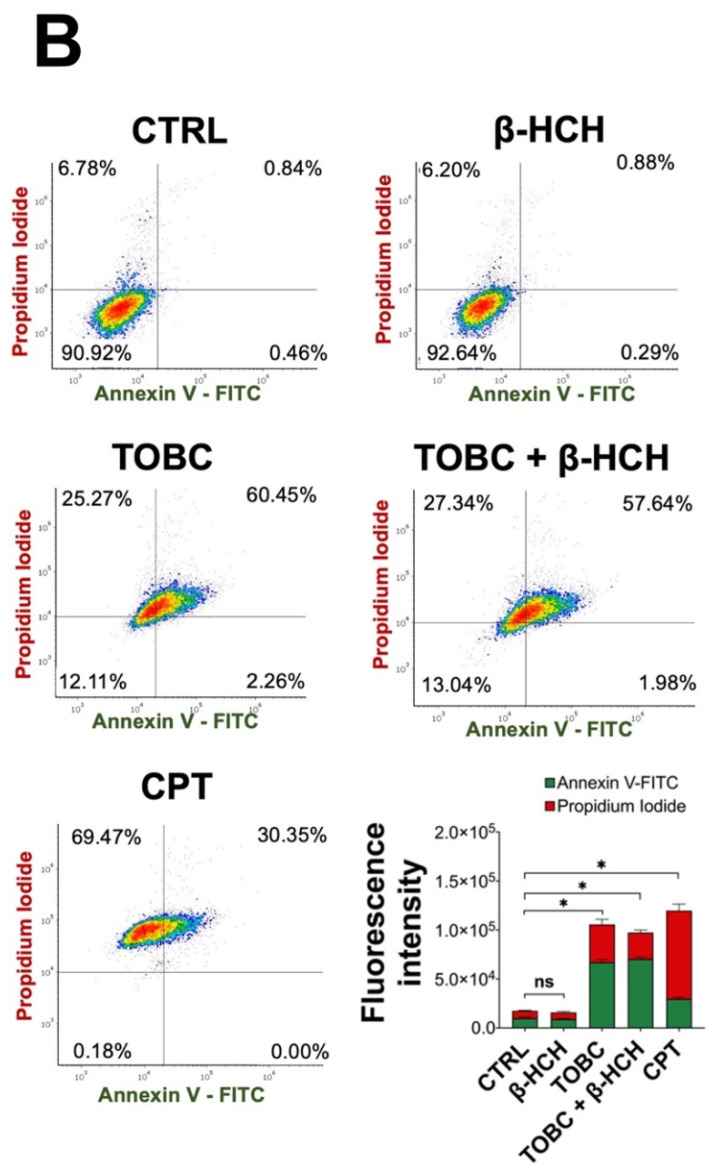
Flow cytometric analysis on LNCaP cells. (**A**) Analyses of cycle distribution evidence that β-HCH induces an increase in percentage of propidium-iodide-stained cells in G2M phase, whereas TOBC counteracts β-HCH proliferative activity, bringing this value back to control level. Experiments were repeated three times with similar results and obtained values are reported as mean and standard deviation. Statistical analysis was performed with GraphPad Prisma software using one-way ANOVA with Dunnet’s post-hoc test. Statistically significant differences were measured between same cell cycle phase (G0G1, S-Phase, G2/M) for each sample and are all referred to control (* *p* < 0.05; ** *p* < 0.005; *** *p* < 0.001; **** *p* < 0.0001); (**B**) Annexin V-FITC assay. While β-HCH alone does not affect apoptotic population, TOBC is able to induce apoptosis in LNCaP cells either untreated or treated with β-HCH. Experiments were repeated three times and analyzed using ModFit LT software with similar results. Values reported in histogram are presented as mean and standard deviation. CTRL: control (untreated cells); β-HCH: cells subjected to 24 h stimulation with 10 µM β-HCH; TOBC: cells subjected to a 3 h stimulation with TOBC 1 mg/mL; TOBC + β-HCH: cells pretreated 3 h with TOBC 1 mg/mL and n for 24 h with 10 µM β-HCH. Statistical analysis was performed with GraphPad Prisma software using Student’s *t*-test. Statistically significant differences (* *p*< 0.05; ** *p*< 0.005; *** *p* < 0.001) are marked with asterisks and are referred to control.

To further corroborate TOBC activity towards β-HCH molecular targets, biochemical assays should be backed up by the assessment of cellular parameters such as cell cycle distribution, apoptosis and clonogenic potential. Flow cytometric evaluation of cell cycle profiles supports the protective value of TOBC. As can be observed in Figure 7A, in fact, LNCaP cells treated with β-HCH displayed an accumulation in G2M phase, which is typical of hyperproliferating cells and a sustained ROS production; this outcome is consistent with the results obtained in our previous study. On the other hand, TOBC appears to counterbalance β-HCH proliferative effect, bringing the distribution in the cell cycle phases back to the control levels.

Evaluation of apoptosis gave some hints on how TOBC can offset β-HCH-induced cell growth. As reported in Figure 7B, flow cytometry analysis revealed that treatment with β-HCH alone did not affect the percentage of apoptotic population, which is significantly increased in samples treated with TOBC or cotreated with TOBC and β-HCH; camptothecin was used as a positive control. Obtained results suggest that TOBC compensates the proliferative activity of β-HCH by inducing apoptosis in LNCaP cells.

### 3.7. TOBC Impairs β-HCH-Dependent Colony Formation

**Figure 8:**
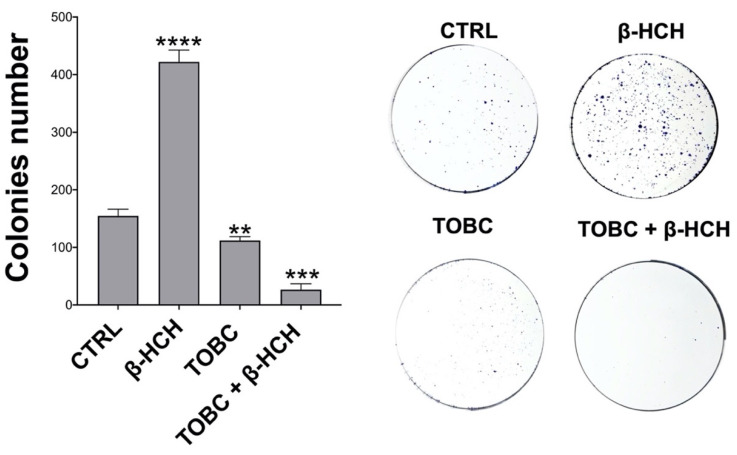
Impact of TOBC on β-HCH-dependent colony formation. Cells were pretreated for one week with 10 μM β-HCH and n for 5 days with 1 mg/mL TOBC. As shown in figure, TOBC contrasts β-HCH-dependent colony formation leading to a decrease in colony number. Experiments were repeated three times with similar results, and obtained values are presented as mean and standard deviation. Statistical analysis was performed with GraphPad Prisma software using Student’s *t*-test. Statistically significant differences (** *p* < 0.005; *** *p* < 0.001; **** *p* < 0.0001) are marked with asterisks and are referred to control.

The clonogenicity assay provides significant information about the differentiation status of a cell population by assessing the capability of a single cell to form a colony. In a previous work, we evidenced the enhanced clonogenic potential induced by β-HCH [18]. To inspect the impact of TOBC on the clonogenicity promoted by β-HCH, LNCaP cells were pretreated for 1 week with β-HCH, and then for 5 days with TOBC. While an increase in colonies number is observable upon treatment β-HCH, a remarkable reduction of colonies is induced by TOBC cotreatment; TOBC alone has a limited effect on clonogenicity (Figure 8). This result is a further proof of TOBC protective function with respect to β-HCH cellular activities.

## 4. Discussion

Environmental contaminants such as OCPs are a major contributor to noncommunicable diseases, with 12.6 million cases of premature deaths annually [67]. Although pollution created great concern and contaminant remediation was the subject of social debate over the past decades, the levels of pollution are continuing to increase, especially in developing countries, and the complete elimination of contamination sources seems not to be a feasible goal in the short-term period. Notwithstanding the urgency to lower the pollution burden, what research could do now to safeguard public health and future generations? To protect the organism against the impact of hazardous substances, an effective approach could be a “human body remediation” strategy that takes into account the molecular basis underlying OCPs action [68]. To address this question, our research group lavished great attention on the study of the biochemical and cellular effects of β-HCH, a globally widespread organochlorine that embodies all the typical physicochemical characteristics of OCPs. Results from our previous studies decrypted the intracellular processes ascribable to β-HCH, identifying some potential biomarkers in a panel of different cell lines. In particular, β-HCH was found to exert its carcinogenicity by means of several mechanisms: modulation of STAT3-mediated oncogenic pathways, AhR activation, disruption of AR signaling cascade, establishment of oxidative stress and induction of genotoxic responses [17,18,19]. The first line of defense against external insults could be a green therapeutic plan providing for the exploitation of natural bioactive compounds to counteract pollutants toxicity [69,70]. Scientific literature gave positive evidence regarding the bioactivity of natural-derived products, which show great structural diversity and elicit a wide range of biological effects. Most of the relevant signaling molecules engaged in the biochemical responses to β-HCH are well-known targets of active principles commonly present in fruits, vegetables and spices; in our investigations, the selection criteria for eligible bioactive compounds encompass cross-referenced data in publicly available databases, with the purpose of drawing a blueprint for natural products able to modulate the activities triggered by β-HCH (Table 1).

**Figure 9:**
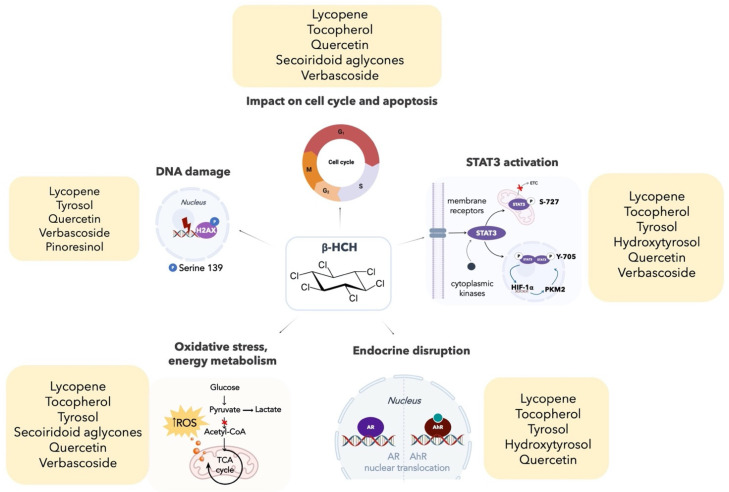
Graphic summary: bioactive principles contained in TOBC solution modulate β-HCH cellular target by means of different mechanisms of action.

The most promising candidates are substances contained in olives and tomatoes which, besides being easy to find components of the Mediterranean diet, exhibit a good specificity for β-HCH cellular targets. The lipophilic solution referred to as TOBC (Tomato and Olive Bioactive Compounds) meets all the requirements to oppose β-HCH negative effects; TOBC, in fact, is obtained from a patented food supplement (patent No. EP2851080A1) enriched with numerous beneficial micronutrients deriving from spray-dried whole tomatoes and olive mill wastewater. Alongside the high nutritional value, TOBC falls within the context of a Green circular economy: the use of waste materials from tomatoes and olives processing allows the recovery of possible contaminant material, contributing to reduce the environmental impact of the entire downstream agri-food chain. In view of these consideration, TOBC was tested on the androgen-sensitive human prostate adenocarcinoma cell line LNCaP, which was already used in our previous studies. The protective role of TOBC was evaluated by looking at the modulatory effects of each of its main components, taken individually, on β-HCH intracellular activities (Figure 9):

(1) lycopene is a well-established antioxidant and STAT3 inhibitor;
(2) tocopherol targets both AR and STAT3 pathways;
(3) tyrosol bioactivity is directed against almost all β-HCH-related biomarkers, from STAT3 to genotoxic responses, from oxidative stress to AR and AhR signaling;
(4) oleuropein is able to reduce the production of ROS;
(5) verbascoside has proven to act on STAT3;
(6) pinoresinol shows antioxidant properties.

The synergic combination of active principles makes TOBC a suitable functional food to be included in a diet regimen aimed at defending cells from β-HCH toxicity. By affecting STAT3-mediated oncogenic pathways and both AR and AhR transduction signaling, TOBC offsets β-HCH-induced proliferative responses and leads to an increase in cell population undergoing apoptotis; the consequent drop in ROS levels, ascribable to the antioxidant components of TOBC, prevents cells from genotoxic damage, as testified by the absence of phosphorylated H2A.X (S139) and the reduction in the extent of DNA fragmentation. The overall TOBC capability of counteracting β-HCH proliferative activity was also reflected in the lower number of colonies measured by the colony formation assay. 

In addition, experiments on the hormone-independent hepatocellular carcinoma cell line HepG2 are currently ongoing to further substantiate the chemoprotective role of TOBC toward β-HCH cellular action; results from the MTT and the wound healing assay reported in Supplementary Material (Figures S2 and S3) indicated the reduced proliferation and migration of HepG2 cells in response to TOBC.

In the final analysis, bioactive compounds contained in TOBC solution can modulate β-HCH cellular targets and represent a valuable ally to develop an easily accessible green nutritional plan, aimed at preventing and combatting health conditions associated with the exposure to environmental pollutants.

## Figures and Tables

**Table 1:** Table displays principal bioactive compounds in 1 mg/mL TOBC solution, indicating their respective concentrations, food sources, and cellular targets.

Bioactive Compound	Concentration	Cellular Targets	Food Sources	Ref.
Tocopherol	40 nM	Apoptosis induction, AR transcriptional inhibition, decreased PSA levels, STAT3 inhibition	Oleaginous fruits, wheat seeds, green leafy vegetables, cereals	[38,39,40,41]
Lycopene	4.6 µM	Decreased PSA levels, antiproliferative activity, apoptosis, reduced clonogenic potential, reduction of the cell number in G0G1 phase, protective role against DNA damage, STAT3 inhibition, antioxidant activity	Guava, watermelon, tomato (cooked), red peppers, papaya, carrots	[42,43,44]
Tyrosol Hydroxytyrosol	200 µM 650 µM	STAT3 inhibition, AhR ligand, decrease in ROS levels, reduced AR expression, decreased PSA levels, protective role against DNA damage, oxidative stress reduction	Olive oil, red wine vinegar	[45,46,47,48]
Quercetin	4.6 µM	STAT3 inhibition, reduced AR expression, decreased PSA levels, protective role against DNA damage, oxidative stress reduction, apoptosis induction, reduced cell proliferation	Tomato, black elderberry, red wine, orange, capers, cloves, almond, onion, dark chocolate	[49,50,51]
Secoiridoid aglycones (oleuropein, ligstroside)	150 µM	Modulation of redox homeostasis, cell apoptosis induction, reduced cell proliferation	Olive oil	[52,53,54,55]
Verbascoside	100 µM	Apoptosis promotion, inhibition of cell migration, STAT3 inhibition, protective role against DNA damage	Olive oil, verbena	[56,57,58]
Pinoresinol	140 µM	Antioxidant, protective role against DNA damage, cell viability decrease.	Olive oil	[59,60]

## Data Availability

Data are contained within the article and the Supplementary Material file.

## Supplementary Materials

The following are available online. Figure S1: Chromatogram from the GC/MS analysis performed to validate the composition of the TOBC solution. Figure S2: MTT assay performed on the hepatocellular carcinoma cells HepG2 pretreated for 3 h with 1 mg/mL TOBC solution (from the batch No. 1265) and then incubated for 48 h with 10 μM β-HCH. Figure S3: Wound healing assay performed on the hepatocellular carcinoma cells HepG2.
