# Supplementary material for: Tomato and Olive Bioactive Compounds: A Natural Shield against the Cellular Effects Induced by β-Hexachlorocyclohexane-Activated Signaling Pathways
Source: Molecules. 2021 Nov 25;26(23):7135. doi: 10.3390/molecules26237135 (PMC8658925; doi:10.3390/molecules26237135)
Supplement: Supplementary file 1 [file molecules-26-07135-s001.zip › molecules-1470746-supplementary.pdf]

Supplementary.

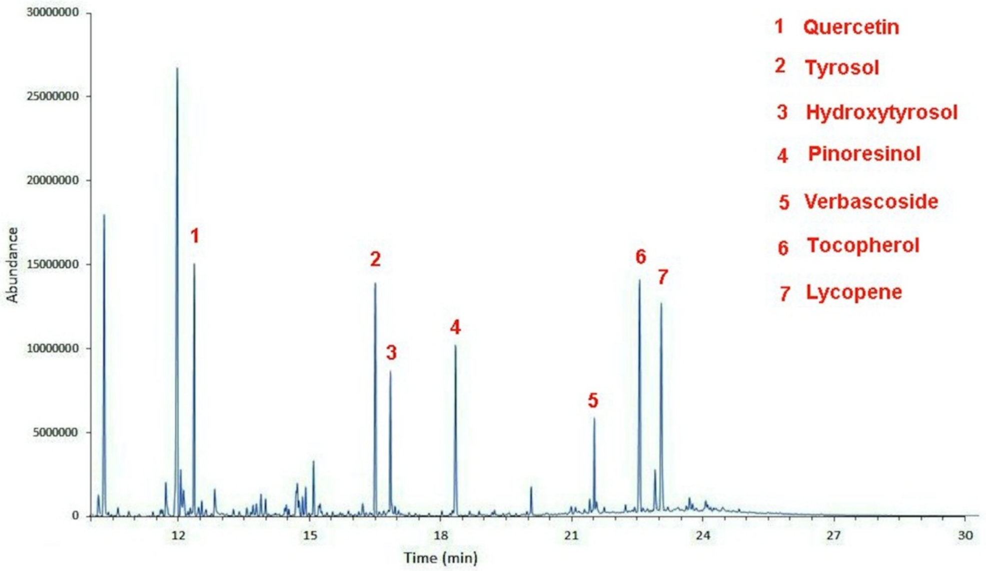

Figure S1. Chromatogram from the GC/MS analysis performed to validate the composition of the TOBC solution.

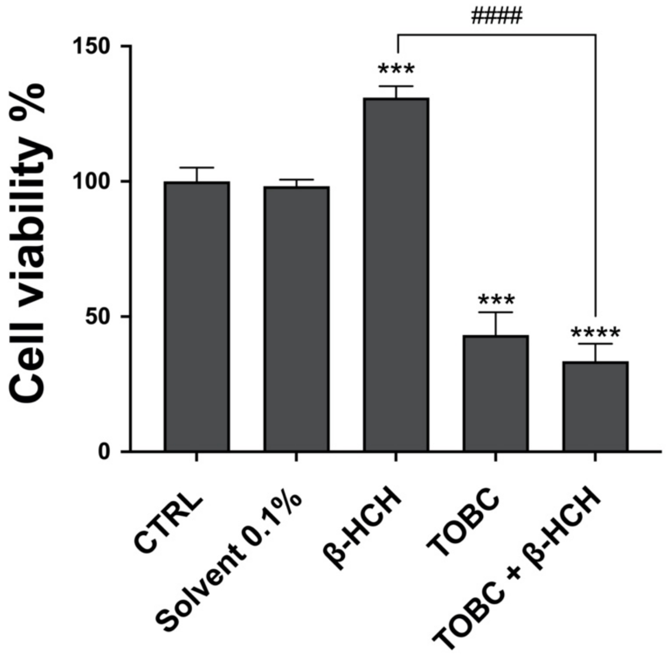

Figure S2. MTT assay performed on the hepatocellular carcinoma cells HepG2 pretreated for 3 hours with 1 mg/mL TOBC solution (from the batch No.1265) and then incubated for 48 hours with 10 μM β-HCH. Experiments were repeated three times with similar results and the obtained values are reported as mean and standard deviation. Statistical analysis was performed with GraphPad Prisma software using Student's t-test. Statistically significant differences referred to the control are marked with asterisks (*** $p < 0.001$; ****$p < 0.0001$); statistically significant differences between β-HCH and TOBC + β-HCH samples are marked with hashtags (####$p<0.0001$).

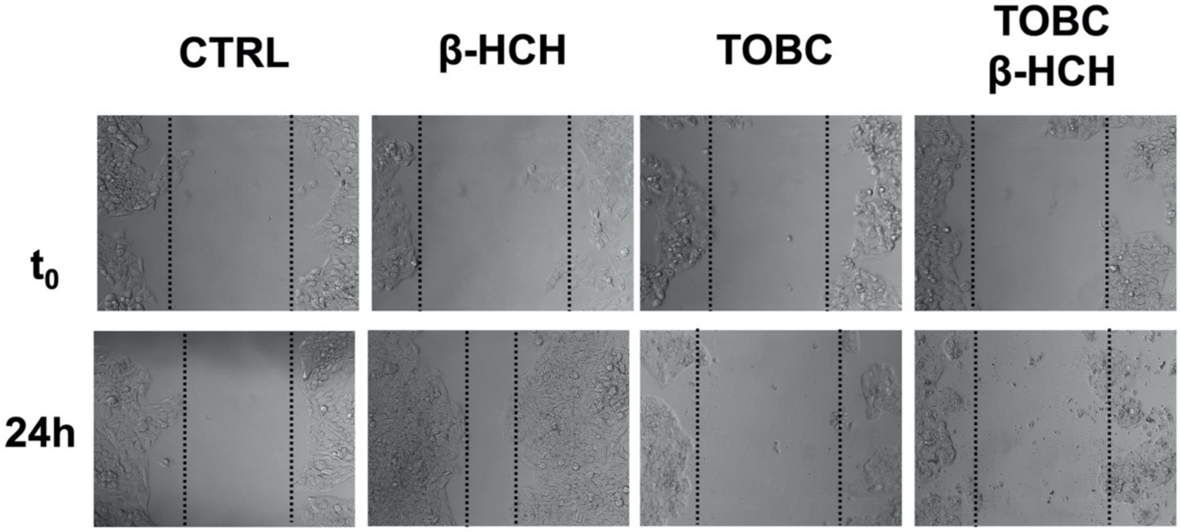

Figure S3. Wound healing assay performed on the hepatocellular carcinoma cells HepG2. Cells were seeded on a 6-wells plate at 150’000 cells/well and a scratch was made after they reached a confluence of 70%. Images corresponding to t_0 were collected immediately after scratching the cell monolayer. HepG2 cells were pretreated for 3 hours with 1 mg/mL TOBC solution obtained from the batch No.1265 and then incubated for 24 hours with 10 μM β-HCH. Cells subjected to 10 μM β-HCH treatment cover the scratch to a larger extent than the untreated sample, whereas cells detachment occurs upon TOBC treatment, either administered alone or in the presence of β-HCH. The panel is representative of three independent experiments.
